# P-1145. Geographic and Genetic Similarities between Sporadic *Haemophilus influenzae* Type b Pediatric Meningitis Cases in New York City

**DOI:** 10.1093/ofid/ofae631.1332

**Published:** 2025-01-29

**Authors:** Anne T Ewing, Caitlin Otto, Adam J Ratner, Sydney Haldeman, Megan Job

**Affiliations:** NYU Grossman School of Medicine, New York, New York; NYU Langone, New York, New York; New York University School of Medicine, New York, NY; New York University Grossman School of Medicine, New York, New York; New York University Grossman School of Medicine, New York, New York

## Abstract

**Background:**

Two unvaccinated infants residing in Brooklyn, NY presented to our hospital with *Haemophilus influenzae* type b (Hib) meningitis within one year of each other. Prior outbreak data suggest that communities with low vaccination rates may have circulation of genetically similar Hib strains. We sought to understand the genomic epidemiology of these geographically related cases, hypothesizing that the strains would be closely related.

**Figure:**
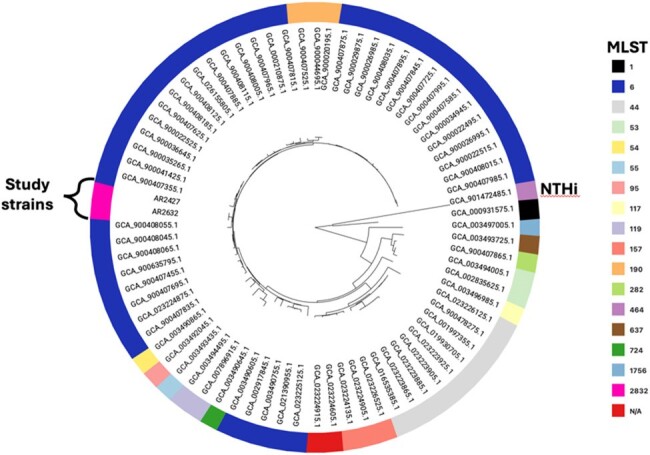

Mash tree of Hib genomes. One non-typeable H. influenzae (NTHi) genome is included as an outgroup. Study cases described are highlighted.

**Methods:**

We performed a retrospective chart review of pediatric patients (0-5 years) seen at our multi-center health system between 2013-2023 with a sterile-site culture positive for Hib. Whole genome sequencing (WGS) was performed on Hib isolates from blood and cerebral spinal fluid (CSF) samples from the 2 identified cases. Multi-locus sequence type (MLST) was determined using the PubMLST database. Single nucleotide polymorphisms (SNPs) and indels were detected with snippy. A genetic distance tree (mashtree) including 75 Hib genomes from GenBank was constructed.

**Results:**

No additional invasive Hib cases were detected over the past 10 years of data. We generated high-quality closed genome sequences from the 2 meningitis cases. Notably, the strains both belonged to a novel MLST, sequence type (ST)-2832, part of the ST-6 complex. Bacterial genetic sequences were identical across sample type (blood vs. CSF) for each patient. Strains from the 2 patients differed at 262 sites (SNPs+indels). A genetic distance tree (Figure) demonstrates that the 2 isolates are more closely related to each other than to any other Hib genomes in the dataset.

**Conclusion:**

The 2 recent meningitis cases represented an increase in incidence of invasive Hib disease at our institution and were both geographically and genetically linked, sharing a novel MLST. Sporadic cases of closely related Hib strains suggest that an under-vaccinated population serves as a reservoir for community strain circulation. Understanding Hib epidemiology can help target public health campaigns, including vaccination efforts.

**Disclosures:**

**All Authors**: No reported disclosures

